# Elemental Ingredients in the Macrophage Cocktail: Role of ZIP8 in Host Response to *Mycobacterium tuberculosis*

**DOI:** 10.3390/ijms18112375

**Published:** 2017-11-09

**Authors:** Charlie J. Pyle, Abul K. Azad, Audrey C. Papp, Wolfgang Sadee, Daren L. Knoell, Larry S. Schlesinger

**Affiliations:** 1 Department of Molecular Genetics and Microbiology, Duke University, Durham, NC 27710, USA; charlie.pyle@gmail.com; 2 Texas Biomedical Research Institute, San Antonio, TX 78227, USA; AAzad@txbiomed.org; 3 Center for Pharmacogenomics, Department of Cancer Biology and Genetics, College of Medicine, The Ohio State University Wexner Medical Center, Columbus, OH 43085, USA; papp.2@osu.edu (A.C.P.); Wolfgang.Sadee@osumc.edu (W.S.); 4 College of Pharmacy, The University of Nebraska Medical Center, Omaha, NE 68198-6120, USA

**Keywords:** zinc, zinc transporter, tuberculosis, lung, macrophage, innate immunity

## Abstract

Tuberculosis (TB) is a global epidemic caused by the infection of human macrophages with the world’s most deadly single bacterial pathogen, *Mycobacterium tuberculosis* (*M.tb*). *M.tb* resides in a phagosomal niche within macrophages, where trace element concentrations impact the immune response, bacterial metal metabolism, and bacterial survival. The manipulation of micronutrients is a critical mechanism of host defense against infection. In particular, the human zinc transporter Zrt-/Irt-like protein 8 (ZIP8), one of 14 ZIP family members, is important in the flux of divalent cations, including zinc, into the cytoplasm of macrophages. It also has been observed to exist on the membrane of cellular organelles, where it can serve as an efflux pump that transports zinc into the cytosol. ZIP8 is highly inducible in response to *M.tb* infection of macrophages, and we have observed its localization to the *M.tb* phagosome. The expression, localization, and function of ZIP8 and other divalent cation transporters within macrophages have important implications for TB prevention and dissemination and warrant further study. In particular, given the importance of zinc as an essential nutrient required for humans and *M.tb*, it is not yet clear whether ZIP-guided zinc transport serves as a host protective factor or, rather, is targeted by *M.tb* to enable its phagosomal survival.

## 1. Introduction

Tuberculosis (TB) is a major cause of global morbidity and mortality. One in three people are infected with the pathogen responsible for TB, *Mycobacterium tuberculosis* (*M.tb*) [1]. Primary infection is established in the lungs, following inhalation of aerosolized respiratory droplets expelled from a contagious person [2]. Infection results in clinical latency in most healthy human hosts but may reemerge as a potentially fatal pneumonia if immune competence is disrupted [3]. *M.tb* is a facultative intracellular bacterium of macrophages that gains cellular entry through phagocytosis and resides within distinctive phagosomes. The successful intraphagosomal survival of *M.tb* is predicated on its circumvention of the mechanisms evolved to destroy phagocytosed pathogens [4]. Macrophage trace element redistribution is a critical host defense strategy against *M.tb* [5].

*M.tb* residence and growth in mononuclear phagocytes depends on its ability to acquire host-derived nutrients within a suitable range. Macrophages and *M.tb* compete for control of elemental cationic micronutrients, which are essential for mycobacterial growth but also toxic at elevated concentrations [5,6,7]. The manipulation of trace element flux is the function of many microbial virulence factors and host immune responses. Due to their charge, cationic micronutrients require specialized transport mechanisms to penetrate the phospholipid bilayers of both the plasma membrane and the phagosome. Infection by *M.tb* alters the battery of membrane spanning ion channels present in macrophages [8,9,10,11]. Clearly a “tug of war” for the control of trace elements between host and microbe exists, and transmembrane spanning metal ion transporters serve as the primary conduit of micronutrient biodistribution during infection. Over the past decade, several examples have emerged and will first be reviewed before a detailed discussion of zinc and zinc transporters.

## 2. Overview of Metal Metabolism at the Host-Pathogen Interface

The term nutritional immunity was coined to describe the anti-microbial benefits associated with redistribution of iron from the vascular space to intracellular compartments [12]. However, it has come to encompass both systemic and cellular nutrient deprivation of multiple trace elements, including iron, manganese, and zinc, from extracellular or intracellular pathogens. A cadre of innate immune effector cells mount that response with the production of trace element binding proteins, cellular importers, and their associated regulatory factors, following pathogen recognition [13]. Macrophages accumulate iron, copper, and zinc during mycobacterial infection [14].

TB is associated with anemia, which results from macrophage iron retention [15]. Although protective against extracellular pathogen growth, iron loading of macrophages may be beneficial to *M.tb* by providing access to essential nutrition. Impaired access to intracellular labile iron reduces the growth of *M.tb* in macrophages from patients with hereditary hemochromatosis [16], a disease that disrupts iron accumulation due to elevated ferroportin-1 (IREG1) export across the plasma membrane [17]. Macrophages have evolved a complex system of intracellular iron redistribution to counter microbial exploitation of cellular iron internalization. The primary mechanism of that defense is the modulation of intraphagosomal iron content. IREG1 is also localized to the mycobacterial phagosome and may serve to sequester iron away from bacteria [9]. Iron is essential for *M.tb* growth [18] and enters the *M.tb* phagosome from intracellular and extracellular stores [19,20]. It can be captured from transferrin or lactoferrin by mycobacterial siderophores, including carboxymycobactins and exochelin, or by heme import [21].

Previously described in detail, macrophage transporters hyper-concentrate trace elements within the phagosome in order to limit mycobacterial growth [5]. Natural resistance-associated macrophage protein 1 (NRAMP1) is a proton/divalent cation antiporter [22] with broad substrate specificity, including iron, zinc, copper, and manganese [23]. Polymorphisms in NRAMP1 are associated with increased susceptibility to pulmonary tuberculosis [24]. In murine models, NRAMP1 is rapidly localized to the phagosome [25,26] and is associated with resistance to intracellular pathogens [27]. NRAMP1 actively acidifies the bacterial phagosome in mice [28]. It increases the translocation of the proton ATPase to the phagosome, following interferon gamma (IFN-γ) activation, leading to the generation of Fenton-mediated free radical production [29,30]. NRAMP1 is capable of shuttling metals bi-directionally against a proton gradient, whereby the direction of transport is determined by proton and divalent cation concentrations [22]. When phagosomal pH and iron levels are lower than those of the cytoplasm, iron is imported into the phagosome through NRAMP1, resulting in the generation of reactive oxygen species (ROS) through the Fenton and Haber-Weiss reactions [7,8]. Alternatively, in instances in which intraphagosomal concentrations are higher than those of the cytosol, NRAMP1 can export iron and manganese and import protons into the phagosome, increasing acidity and depriving pathogens of those essential nutrients [30,31].

Macrophages also use a strategy involving the phagosomal hyper-concentration of copper [14] through a separate set of copper transporters [32]. IFN-γ activation of murine macrophages induces the expression of the plasma membrane copper transporter CTR1, leading to copper uptake. Subsequent translocation of the copper importer ATP7A to mycobacterial phagosomes leads to increased intraphagosomal concentrations of copper and thereby generation of bactericidal Fenton free radicals [10,33]. As a countermeasure, *M.tb* actively up-regulates the mycobacterial copper transport protein B (MctB), which rescues it from copper toxicity [34].

## 3. Regulation of Zinc Balance between Host and Pathogen

The transient hyper-accumulation of zinc in the phagosomes of human macrophages reduces the survival of phagocytosed extracellular pathogens. Zinc accumulation causes the up-regulation of bacterial cation efflux pumps, which are critical for the adaptation of intracellular pathogens, including *Salmonella typhimurium* and *M.tb* [35,36]. The mycobacterial manganese efflux pump Metal cation-transporting p-type ATPase C (CtpC) is required for *M.tb* survival at high zinc conditions [37]. Although no definitive mechanism for zinc-associated toxicity has yet been identified in these models, there are several potential avenues through which elevated zinc concentrations may be toxic to *M.tb*. Those mechanisms include the displacement of iron from sulfhydryl moieties of bacterial enzymes [38] or the disruption of manganese uptake, which reduces bacterial free radical tolerance [39]. The mycobacterial transcriptional repressors Zur and IdeR sense elevations in zinc and iron, respectively, leading to reduced expression of the gene cluster for the type VII secretion system 6 kDa early secretory antigenic target protein family secretion system-3 (ESX-3) [40,41]. Metal-dependent suppression of the critical mycobacterial virulence factors EsxG and EsxH in the ESX3 locus thereby reduces *M.tb* survival [42].

The mechanism through which zinc traverses macrophage membranes in response to *M.tb* infection remains an area of active investigation. Twenty-four dedicated zinc transport proteins are primarily responsible for zinc biodistribution. Each transporter has distinct induction patterns, expression profiles, subcellular localization, and tissue distribution, providing each transporter with a unique role in zinc metabolism. Ten solute carrier 30A (SLC30A) family members, the zinc transport proteins (ZnTs), remove zinc from the cytoplasm across the plasma membrane or into cytosolic organelles. Conversely, fourteen solute carrier 39A (SLC39A) family members, the Zrt-Irt-like-Proteins (ZIPs) move zinc into the cytoplasm from the extracellular environment or out of intracellular vesicles [43,44]. Individually, some ZIP and ZnT proteins have been shown to traffic other divalent cations [45,46,47]. Further, other nondedicated divalent cation transporters have the capacity to transport zinc, including NRAMP1, IREG1, and divalent metal transporter 1 (DMT1) [23,48,49].

**Figure 1:**
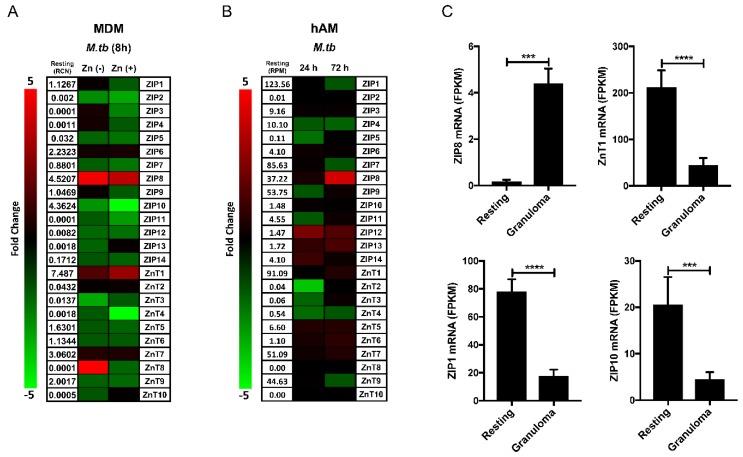
Macrophage zinc transporter mRNA expression during *M.tb* infection. Zrt-Irt-like-Protein (ZIP) and zinc transport protein (ZnT) mRNA expression is altered by infection with *Mycobacterium tuberculosis* (*M.tb*) H_37_R_v_ using a multiplicity of infection (MOI) of 5:1 for (**A**) 8 h in monocyte-derived macrophages (MDMs) in the absence or presence of ZnSO_4_ 18 μM, as determined by qRT-PCR relative to GAPDH (*n* = 3) or (**B**) for 24 or 72 h in human alveolar macrophages (hAMs) infected by *M.tb* H_37_R_v_, as determined by AmpliSeq Transcriptome analysis (*n* = 6). (**C**) The mRNA expression of ZIP8 is increased and ZnT1, ZIP1, and ZIP10 are decreased in *M.marinum*-infected zebrafish granulomas compared to resting macrophages, as determined by RNA-Seq. (A and B are unpublished data; C was generated using supplementary data published in Cronan et al. [53]) (mean ± SEM; *** *p* < 0.001; **** *p* < 0.0001; Prism-7: one-tailed Students *t*-test). MDM [54] and hAM [55] isolation, culture, and infection with *M.tb*, as well as the assay of human zinc transporters by qRT-PCR in MDMs [56] and AmpliSeq Transcriptome analysis in hAMs [52], were performed as previously described.

There is constitutive mRNA expression above a threshold of one relative copy number (RCN), for ZIPs 1, 6, 8, and 10, as well as ZnTs 1, 5, 6, 7, and 9, in resting human monocyte-derived macrophages (MDMs). The infection of MDMs with virulent *M.tb* H_37_R_v_ for 8 h alters the expression pattern of several ZIPs and ZnTs, indicating a global disruption of zinc homeostasis (Figure 1A). In RPMI media supplemented with autologous human serum, infection does not significantly alter the expression of ZIP1 or 6 but decreases ZnTs 5, 6, and 9 and slightly increases ZnT7. ZnT7 expression has previously been shown to increase in response to infection by intracellular fungal pathogens [50]. The resting expression of ZnT1, ZIP8, and ZIP10 is the highest among the 24 zinc transporters. *M.tb* infection significantly increases ZnT1 and ZIP8 but decreases ZIP10. Although infection alters the expression of multiple ZIPs and ZnTs, ZIP8 is unique as it is the sole ZIP zinc importer induced by *M.tb* in MDMs. Human alveolar macrophages (hAMs) reside within a unique microenvironmental niche in the alveolus (gas exchange apparatus) and are the phagocytic cells initially targeted by *M.tb* during airborne infection [51]. As might be expected, the resting expression pattern of zinc transporters in hAMs varies substantially from that of MDMs. There is constitutive mRNA expression above a threshold of 10 reads per million (RPM), determined as described [52], for ZIPs 1, 4, 7, 8, and 9, as well as ZnTs 1, 7, and 9 (Figure 1B). The infection of hAMs with *M.tb* H_37_R_v_ also alters ZIP and ZnT mRNA expression. As compared to ZIP8 in MDMs, ZIP1 is the most highly expressed ZIP in resting hAMs, and its expression is reduced following 72 h of *M.tb* infection. Additionally, infection results in the reduced expression of ZIPs 4 and 9 within 24 h, as well as the reduced expression of ZIP7 and ZnT9 after 72 h. ZIP8, ZnT7, and ZnT1 are increased during infection. Although there is a comparative delay in the induction of ZIP8 expression in hAMs, it again emerges as the most responsive zinc transporter to *M.tb* infection. The alterations we observed in ZIP and ZnT expression in each of our in vitro models likely impact zinc metabolism within particular macrophage subsets in distinct ways, with each transporter contributing to specific aspects of cumulative cellular zinc flux. The expression profile of ZIP8 is unique as it has high constitutive expression and is the most responsive zinc transporter to *M.tb* infection in MDMs and hAMs. ZIP8 is the only zinc importer increased by *M.tb* in MDMs and emerges as the dominant ZIP expressed in hAMs during infection, although ZIPs 12, 13, and 14 do increase from low resting levels in that model. Overall, the stimulation of ZIP8 expression by *M.tb* is a prominent feature of the macrophage response to infection, which should be viewed in the context of a generalized shift in cellular zinc metabolism.

In an important study, Botella et al. [36] revealed that *M.tb* infection of human macrophages induces mRNA expression of metallothionein (MT) intracellular zinc binding proteins and ZnT1 by activating the metal responsive transcription factor MTF-1. Based upon these observations, a model of ZnT1 phagosomal localization as a mechanism for the hyper-accumulation of vesicular zinc was proposed [36]. The contribution of phagosomal NRAMP1 relative to increases in zinc should also be considered [23,25]. The activation of MTF-1 and subsequent MT transcription indicates that zinc levels also increase within the cytosolic compartment during *M.tb* infection. In cell culture models with limited extracellular zinc, MTF-1 transcriptional activation by zinc is likely exclusively due to intracellular redistribution [36]. Knowing that cellular zinc trafficking within the human physiologic range, both high and low, is an essential aspect of macrophage metal metabolism, future studies of zinc flux across the plasma membrane during *M.tb* infection should include zinc levels that simulate the human condition. Physiologically relevant zinc supplementation [56] of *M.tb*-infected MDMs in vitro alters their mRNA expression profile. The addition of zinc during infection results in the reduced expression of most zinc transporters (Figure 1A). It reduces the extent of ZIP8 induction but increases ZnT1 expression and leads to further repression of ZIP1 and ZIP10 in response to infection. Cumulatively these changes indicate a shift toward cytosolic zinc efflux. The increased transcriptional activation of ZnT1 [57,58], coupled with the suppression of ZIP10 expression [59] during supplementation, indicates that the extent of MTF-1 activation in infected macrophages is dependent on extracellular zinc import.

In a recent study using the zebrafish model of TB pathogenesis, Cronan et al. evaluated the impact of mycobacterial reprogramming of granuloma macrophages and provided a granuloma-specific macrophage transcriptomic signature [53]. Parsing of that data revealed that ZIP8 is highly induced and that ZIP1 and ZIP10 expression is reduced in mycobacterial granulomas (Figure 1C). Surprisingly, ZnT1 expression is significantly reduced in granuloma macrophages, which may reflect altered zinc metabolism during mesenchymal-epithelial transition or species-specific differences. However, ZIP8 induction during mycobacterial infection appears to be a critical, evolutionarily conserved response that is maintained during granuloma formation and among the lineages of human macrophages that are central to TB pathogenesis (Figure 1A,B).

## 4. ZIP8 in Macrophage Infection by *M.tb*

**Figure 2:**
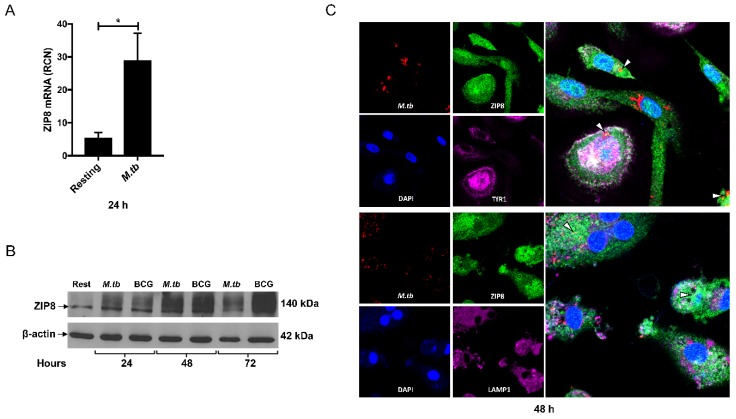
ZIP8 protein is induced and localizes with *M.tb* in human macrophages. MDM production of ZIP8: (**A**) mRNA is significantly induced for 24 h, following infection with *M.tb* H_37_R_v_ (MOI 5:1), as determined by qRT-PCR relative to GAPDH (*n* = 3) and (**B**) ZIP8 protein is robustly increased by infection with *M.tb* H_37_R_v_ or *M.bovis* BCG (MOI 5:1) between 24 and 72 h, as determined by Western blot relative to β-actin (*n* = 3). (**C**) The infection of MDMs with mCherry expressing *M.tb* H_37_R_v_ (MOI 5:1) for 48 h leads to the extensive co-localization of ZIP8 with *M.tb* (yellow; indicated by arrow heads) and TfR1 (abundant white; in merged upper panel) but very limited co-localization with LAMP-1 (negligible white; in merged lower panel) (A, B, and C are unpublished data) (mean ± SEM; * *p* < 0.05; Prism-7: one-tailed Students *t*-test). MDM isolation, culture, and infection [54]; qRT-PCR and Western blot of ZIP8 in MDMs [56]; and confocal fluorescence microscopy using an Olympus FV1000-Spectral System at 60× magnification in infected MDMs [67] were performed as previously described. Rabbit polyclonal antiserum anti-peptide to amino acid residues 225 to 243 of human ZIP8 was purchased from Covance (Princeton, NJ, USA). Mouse anti-human monoclonal β-actin (#69101) antibody was purchased from MP Biomedicals (Santa Ana, CA, USA). Mouse anti-human monoclonal CD71 (#334102) antibody was purchased from Biolegend (San Diego, CA, USA). Mouse anti-human monoclonal LAMP-1 (#ab25630) antibody was purchased from Abcam (Cambridge, UK).

The manipulation of intracellular zinc through altered macrophage zinc transporter expression impacts the growth of intracellular yeast [60,61], fungal [50,62], and bacterial [35] pathogens. Zinc redistribution into the bacterial phagosome is Tol-like receptor (TLR)-dependent [35]. TLR4 activation of human macrophages induces the production of ZIP8 through Nuclear factor kappa-light-chain-enhancer of activated B cells (NF-κB) [63], and *M.tb* infection transiently induces NF-κB [55,64]. ZIP8 was discovered due to its production in human monocytes in response to *Mycobacterium bovis* Bacillus Calmette Guérin (BCG) cell wall cytoskeletal extract and BCG infection [65]. It is expressed in human macrophages in response to infection with nonpathogenic and virulent mycobacterial species, including *M.tb*, as well as to gram-negative and gram-positive bacteria [11]. In particular, MDM infection with virulent *M.tb* H_37_R_v_ results in the robust induction of ZIP8 mRNA for at least 24 h post infection (Figure 2A). Consistent with our previously published results involving other cell types [66], ZIP8 induction results in the production of a membrane bound, glycosylated 140 kDa protein. ZIP8 protein in MDMs is elevated within 24 h, following infection with *M.tb* H_37_R_v_ or BCG, and remains elevated for at least 72 h (Figure 2B).

Cytosolic zinc import in activated macrophages is ZIP8-dependent [56]. ZIP8 is present on the plasma membrane and on intracellular vesicles in primary human macrophages, epithelial cells, T-cells, and cell lines [63,65,66,68,69], indicating a role for ZIP8 in cytosolic cation increase through cellular influx and vesicular efflux. Viral induced over-expression of ZIP8 in murine chondrocytes increases MTF-1 nuclear localization and transcriptional activity [70]. Slc39a8 hypomorphic mouse fetal fibroblasts have reduced MT expression in response to tumor necrosis factor alpha (TNFα) [63], indicating that ZIP8-dependent zinc increases the transcription of MTF-1 target genes. Furthermore, ZIP8 regulates ZnT1 expression in primary human macrophages [56]. Based upon these observations, it is plausible that macrophage zinc loading during *M.tb* infection [14] is a function of zinc import through ZIP8, which then leads to observed elevations in macrophage MT and ZnT1 mRNA expression through the activation of MTF-1.

ZIP8 has multiple glycosylation sites and potential protein-binding partners that may influence membrane orientation and localization [66,71,72]. Further, zinc deprivation of the bacterial phagosome in macrophages and dendritic cells through the up-regulation and trafficking of ZIP8 to the phagosome-lysosome pathway has been proposed [73]. Knowing this, we determined the cellular localization of ZIP8 [63] in relation to early phagosome marker transferrin receptor-1 (TfR1), late endosome/lysosome marker LAMP-1, and *M.tb* in macrophages by fluorescence confocal microscopy, as previously described [67]. We observed that ZIP8 becomes abundant within the phagosome and co-localizes with *M.tb* (Figure 2C). Further, the association of ZIP8 and the pathogen is durable and persists within the phagosome over an extended time frame. Co-localization studies with TfR1 and LAMP-1 indicate that ZIP8 resides primarily within the phagosome, akin to TfR1, and not the phagolysosome in macrophages (Figure 2C). In consideration of the zinc-poisoning paradigm that was previously highlighted, this result indicates a potential role for ZIP8 in both macrophage zinc loading and eventual phagosomal detoxification, following the initial super-concentration of vacuolar zinc that has been observed within 24 h of infection [14,36]. The coordination of zinc efflux through the paired induction of *M.tb* CtpC and macrophage ZIP8 expression may generate a complimentary safeguard in favor of *M.tb* against zinc poisoning. In this context, the induction of ZIP8 expression during *M.tb* infection may serve as a host susceptibility factor.

Alternatively, it is important to consider that all microbes require zinc for survival [74] and that zinc at appropriate concentrations enhances mycobacterial growth [75]. Zinc has the capacity to interact with many proteins in both eukaryotic and prokaryotic cells. The antioxidant properties of zinc afford the protection of vulnerable sulfhydryl groups from damage by ROS [76], which are akin to those generated by high phagosomal concentrations of iron [8] or copper [34]. Thus, high phagosomal zinc concentrations may actually benefit *M.tb* in some regards by enhancing access and limiting the damage incurred from free radical production by other trace elements. Therefore the ZIP8-dependent sequestration of zinc away from *M.tb*, as with intracellular fungal pathogens [50,62], may actually have some host protective effects.

**Figure 3:**
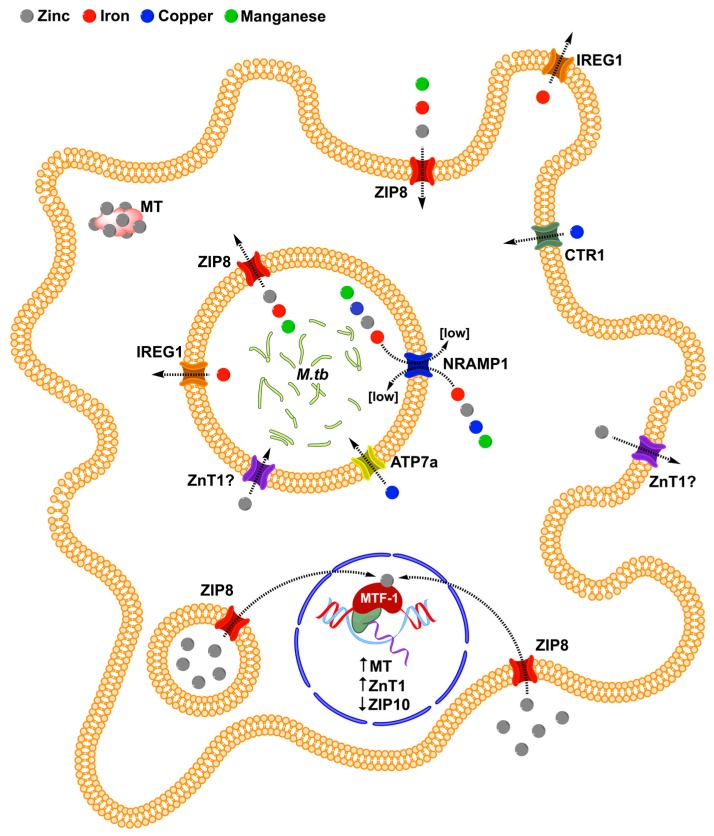
The hypothetical impact of ZIP8 on MTF-1 expression, as well as the localization and direction of trace element transport in macrophages during early infection with *M.tb*. Arrows indicate the direction of trace element transport.

Cation transport by ZIP8 is pH dependent and electroneutral, indicating that it facilitates the co-transport of other ionic species as well [47,69,71]. ZIP8 participates in the cytosolic influx of manganese, cadmium, iron, zinc, and selenite [71,77]. Iron and zinc inhibit the ZIP8-mediated uptake of each other [69]. ZIP proteins contain binuclear metal centers, where metal binding at one site affects the transporter metal selectivity at the second site [78]. Zinc uptake by ZIP8 is competitively inhibited by both iron and cadmium and non-competitively by cobalt, nickel, and copper but is not inhibited by magnesium or manganese [79]. Given that ZIP8 has a relatively high substrate promiscuity, along with a directional transport and localization profile, it is reasonable to expect that, in combination with other metal transporters, ZIP8 contributes to the flux of multiple divalent cations toward and or away from *M.tb* across a number of macrophage membranes, including the mycobacterial phagosome (Figure 3).

ZIP8 and NRAMP1 share multiple substrates, raising the possibility that there may also exist dynamic interplay between the two transporters on the *M.tb* phagosome for the regulation of iron and zinc. Further, iron and zinc within the phagosome may antagonize the transport of one another in a similar way to what occurs in the intestine [80]. ZIP8-dependent iron transport across the phagosomal membrane has the potential to contribute to the previously proposed models of phagosomal iron deprivation [15], involving other transporters such as NRAMP1 [30] or IREG1 [9]. That efflux could counteract the host protective iron-dependent generation of intraphagosomal ROS [8]. ZIP8 activity is pH dependent and potentially drives bicarbonate flux [46]; therefore, it may also impact intraphagosomal pH, which is critical to the maintenance of the intracellular mycobacterial niche [81]. Ultimately the impact of ZIP8 on mycobacterial growth and survival within the *M.tb* phagosome depends on a complex array of variables, including but not limited to host nutritional status and genetic variation [82,83,84], and the co-expression and localization of other trace element transporters, as well as mycobacterial responses to metal flux.

## 5. Implications of ZIP8 Induction on TB

ZIP8 expression is highly induced in human and murine tissues and circulating cells during systemic and local inflammation [70,85,86,87]. The increased intracellular sequestration of zinc, similar to iron, is a mechanism of nutritional immunity in which vascular trace element deprivation limits the extracellular growth of invading pathogens [12]. Zinc, as a vital commodity in times of need, is also therefore redirected to biosynthetic processes that bolster host immune defense. Zinc mobilization from the vasculature into the vital organs predominantly occurs due to ZIP14 induction in the liver [88]. Our group and others have shown that several ZIPs are involved in zinc redistribution into vital organs other than the liver, which is in part due to sequestration of zinc folowing ZIP8 [63], as well as ZIP14 [89] induction in tissue macrophages. ZIP8 is elevated in circulating peripheral blood monocytes during the acute phase response, which is associated with reduced serum zinc concentrations [86]. Similarly, circulating levels of zinc in plasma or serum are reduced in patients with active TB [90,91,92]. In time, zinc levels recover with antibiotic therapy in the absence of zinc supplementation [90,93], indicating that, similar to iron, the intracellular redistribution of vascular zinc occurs in TB. Based on this and our findings, we propose that the ZIP8-dependent iron and zinc loading of macrophages is likely a driving force in systemic nutritional immunity during active TB.

A balance between pro-inflammatory and immune suppressive cytokines is critical for the control of *M.tb* [94,95]. ZIP8 activity modulates the inflammatory response as an intracellular second messenger, thereby altering the production of pro-inflammatory cytokines and IL-10 [56,63,68,70]. IL-10 is an important immune modulator during *M.tb* infection. It is associated with TB progression [96] and reactivation [97] in murine models and reduced macrophage host defense capabilities [98,99,100] or, alternatively, may enhance the control of the infection [101]. ZIP8-dependent zinc import skews cytokine signaling towards a pro-inflammatory profile in activated macrophages, particularly through the inhibition of IL-10 production [56]. Conversely, in activated monocytes, ZIP8 induction leads to negative feedback inhibition of NF-κB and the reduction of pro-inflammatory cytokines [63] that, at balanced levels, are critical for the control of *M.tb* [4]. Further investigations are needed to clarify the impact of ZIP8 on cytokine responses in TB.

Observations that *M.tb* infection increases macrophage intracellular zinc accumulation, leading to MTF-1 nuclear localization and transcriptional activity 24 h after infection [36], are likely due, in part, to ZIP8-dependent zinc influx. In a murine *Slc39a8* floxed; *Col2a1-Cre* chondrocyte-specific ZIP8 knockout model, ZIP8-dependent zinc increased intracellular zinc concentrations and the MTF-1 dependent transcription of matrix metalloproteinases (MMPs) during inflammation [70]. Subsequent MMP activity resulted in the degradation of the surrounding tissue. *M.tb* infection induces MMP production by macrophages and surrounding cells, which results in pulmonary tissue destruction [102,103]. In animal models, during the early phase of mycobacterial granuloma formation, MMP release enhances macrophage recruitment to the site of infection [104], which is associated with increased macrophage infection and dissemination [105]. Furthermore MMP catalytic activity and function requires zinc [106]. The antibiotic doxycycline is the only known Food and Drug Administration-approved MMP inhibitor and has been suggested as an adjunct antibiotic therapy because it reduces *M.tb* growth in vitro and in vivo [107]. In light of these findings, it is intriguing to speculate that ZIP8 induction and subsequent zinc influx increases susceptibility to *M.tb* by driving the MTF-1 transcription of MMPs, resulting in tissue destruction, increased macrophage recruitment, and bacterial growth.

ZIP8 is an interesting target for host-directed pharmacotherapy for the treatment and prevention of TB. To our knowledge, there are no specifically targeted pharmacological antagonists or agonists of ZIP transporters currently available. If the induction of ZIP8 is confirmed to be a pathogenic process during mycobacterial infection, the development of small molecule inhibitors or biopharmaceuticals could prove valuable. However, within the framework of global zinc homeostasis, the selective targeting of ZIP8 may have myriad effects. Alternatively, a strategy of zinc supplementation in an effort to nullify the potential antagonism of zinc poisoning by phagosomal ZIP8 could have some utility as a preventative therapy for high risk populations, although, to date, zinc supplementation has not been shown to have clinical benefit during active TB [108].

## 6. Conclusions

Tuberculosis is the world’s most deadly infectious disease caused by a single pathogen. Globally it is estimated that over 10 million people are infected with *M.tb* every year. Unfortunately, TB treatment is limited by lack of universal availability of effective medications, drug toxicity, and lack of compliance, the last resulting in the increasing incidence of multiple drug resistant strains. This is further complicated because individuals with chronically depressed immunity have a much higher risk of developing TB [1]. Zinc deficiency is a major cause of immune dysfunction and infection [109]. *M.tb* infection influences human zinc metabolism. Understanding the complexities of macrophage responses to *M.tb* is particularly important, given its substantial impact on global human health. An alteration in the trafficking of divalent cations is an established host defense strategy against extracellular and intracellular pathogens, including *M.tb*. Multiple metal binding proteins and transporters contribute to those responses. ZIP8 is induced in response to *M.tb* infection and localizes to the plasma membrane and intracellular vesicles, including the *M.tb* phagosome. In concert with other established cation transporters, ZIP8 may be positioned in the phagosome to render a fundamentally important impact on macrophage host defense and TB pathogenesis. Further, ZIP8 has been shown to have potent immunomodulatory functions that are influenced by systemic and cellular zinc status. The impact of zinc metabolism on macrophage host defense functions during *M.tb* infection remains an underexplored area of research with promising potential for the generation of translationally applicable future findings.

## Abbreviations

TB	Tuberculosis
*M.tb*	*Mycobacterium tuberculosis*
ZIP	Zrt-/Irt-like protein
IREG1	Ferroportin-1
NRAMP1	Natural resistance-associated macrophage protein 1
IFN-γ	Interferon gamma
ROS	Reactive oxygen species
CTR1	High affinity copper uptake protein-1
ATP7A	ATPase copper transporting alpha
MctB	Mycobacterial copper transport protein B
CtpC	Metal cation-transporting p-type ATPase C
Zur	Zinc uptake repressor
IdeR	Iron-dependent repressor
ESX-3	6 kDa early secretory antigenic target protein family secretion system-3
EsxG	ESAT-6-like protein G
EsxH	ESAT-6-like protein H
ZnT	Zinc transport protein
DMT1	Divalent metal transporter 1
MDM	Monocyte-derived macrophage
hAM	Human alveolar macrophage
MT	Metallothionein
MTF-1	Metal responsive transcription factor-1
TLR	Tol-like receptor
NF-κB	Nuclear factor kappa-light-chain-enhancer of activated B cells
BCG	*Mycobacterium bovis* Bacillus Calmette Guérin
kDa	kiloDalton
qRT-PCR	Quantitative Real-Time polymerase chain reaction
MOI	Multiplicity of infection
TfR1	Transferrin receptor-1
LAMP-1	Lysosome-associated membrane protein-1
TNFα	Tumor Necrosis Factor alpha
IL-10	Interleukin 10
MMP	Metalloproteinase
